# Increase of malaria attacks among children presenting concomitant infection by *Schistosoma mansoni *in Senegal

**DOI:** 10.1186/1475-2875-3-43

**Published:** 2004-11-15

**Authors:** Cheikh Sokhna, Jean-Yves Le Hesran, Pape A Mbaye, Jean Akiana, Pape Camara, Mamadou Diop, Abdoulaye Ly, Pierre Druilhe

**Affiliations:** 1 UR Paludisme afro-tropical, IRD, Dakar, Sénégal; 2 UR Santé de la mere et 1'enfant en milieu tropical, IRD, Dakar, Sénégal; 3 Région médicale de Saint Louis, Programme de lutte contre la bilharziose, Sénégal; 4 District médical de Richard-Toll, Sénégal; 5 Unité de Parasitologie BioMédicale, Institut Pasteur, 28, rue du Dr Roux, 75015 Paris, France

## Abstract

Helminthic infections concomitant with malaria are common in inter-tropical areas. A recent study showed that mice co-infected with *Schistosoma mansoni *and *Plasmodium chabaudi *develop higher *P. chabaudi *parasitaemia and had a higher mortality rate. This important observation deserved to be further investigated among human populations.

Malaria attacks were recorded in 512 children aged 6–15 years living in Richard Toll (Northern Senegal) among whom 336 were infected by *S. mansoni*, and 175 were not. The incidence rate of malaria attacks was significantly higher among *S. mansoni*-infected individuals, particularly those carrying the highest worm loads, as compared to uninfected subjects (26.6% versus 16,4 %). In contrast, the rate of malaria attacks was lower, without reaching significance, in medium grade *S. mansoni *infections. Thus, infection by *S. mansoni *affects susceptibility to malaria, but this can vary according to the intensity of parasite load. The immunological mechanisms underlying this dual effect need to be further explored.

## Introduction

Malaria is prevalent in many parts of tropical Africa, where other concomitant parasitic infections are common. However, little is known about how concurrent infections affect the expression to and/or pathogenesis of each other. A recent study showed that *Schistosoma mansoni *and *Plasmodium chabaudi *co-infected mice develop higher *P. chabaudi *parasitemia and higher mortality rate [1]. This observation was supported in the case of human infections in subjects carrying intestinal helminths who were given Levamisole which led to the reduction of the malaria attack rate [2] and by further studies in helminth-free and helminth-carrying malaria-exposed individuals, which confirmed the deleterious effect of worm carriage upon an individual's susceptibility to malaria [3].

In the Senegal river basin, where malaria is hypoendemic, extensive irrigation programmes have been developed, promoting a spread of *S. mansoni *infection [4]. The exposure of the inhabitants to these two infections, in a situation where other helminthic infections were rare, provided an opportunity to study the expression of malaria in a schistosoma exposed population.

## Patients and methods

### Study area

The study took place in Gallo Malick, a district of Richard Toll, which is located near a canal ensuring the irrigation of rice and sugar cane fields. The population was estimated in 1998 to reach 3,685 inhabitants. Annual rainfall amounts to around 250 mm water. The district is approximately 1 km long and 500 m wide. A dispensary is set up in the centre of the district and is thus easily accessible.

### Population study

In September 1998, after obtaining agreement from the parents, two stools examinations were carried out in all children aged between 5 and 15 years. From 25 mg of stools, two slides were prepared, according to the modified Kato-Katz method [5]. The parasitic burden was obtained by multiplying the average value of the burden of the two slides by 40 to express the result in the number of eggs per gram of stools. Children were given a card that entitled them to have free access to the health centre. For each consultation motivated by an episode of fever or an history of fever within 24 hours, a thick smear was performed. Thick smears prepared from capillary blood were examined over 200 microscopic fields. The average number of leucocytes per field was estimated in 10 fields. Parasite density was evaluated based on an average 8,000 leukocytes per μl of blood. Following national recommendations, any fever attack was considered suspect of malaria and treated by chloroquine (25 mg/kg over three days in a 10-10-5 posology), as the drug which was still effective in this area at that time.

The follow-up was implemented between September 1998 and April 1999. In January 1999, a further stool examination and a urine filtration for the detection of *Schistosoma haematobium *eggs, were performed. Ten ml of urine were filtered through a 12 μ millipore membrane and the eggs on the whole filter surface were counted. A questionnaire was issued to check habits with regard to protection against mosquitoes and usage of anti-malarial drug-prophylaxis. It was also ascertained that none of the included children had been administered anti-*S. mansoni *treatment (praziquantel) over the past year (September 97 – September 98). In March 99, all children who presented with at least one *S. mansoni *positive stool examination were treated with praziquantel (40 mg/kg).

Any child presenting with a body temperature > 38°C or having a history of fever-in the 24 hours preceding the consultation and with a parasite density ≥ 5000 parasites/μl of blood was defined as suffering from a malaria attack.

Any child whose tool tested positive for *S. mansoni *at least once was defined as *S. mansoni *positive. If both examinations proved positive, the highest parasitic burden was kept for further analysis. Parasite loads were classified into four categories: A) 1 to 100, B) 101 to 400, C) 401 to 1000, and D) > 1000 eggs per one gram of stools.

In order to exclude possible interactions with other helminthic infections, children from the group not infected by *S. mansoni *but presenting other intestinal parasitic infection were excluded from the analysis.

When houses where children had a malaria attack and those where the children were carrying schistosomiasis were located on a map, the two pathologies were found to be distributed in a uniform way in the whole district, without evidence of clustering of double infection malaria-*S. mansoni*. This is in agreement with the distinct sources of infection, *Anopheles *larvae breeding in small water collections in gardens, and *Schistosoma *vectors in rice fields and irrigation canals.

Comparison between children presenting at least one malaria attack and children not having presented any malaria attack was performed using a forward logistic regression model using STATA statistical software. The variables included in the model were sex, *S. mansoni *categories eggs load and age.

The period between the beginning of the study and the date of a malaria attack was compared between groups of *S. mansoni *infections using a lifetime estimate table from the EGRET programme (Statistics and Epidemiology Research Corporation, Seattle, Washington). Any p value lower than 0.05 was considered significant.

## Results

In September 1998, 525 children underwent two stool examinations. Three-hundred and thirty-six-were found to carry *S. mansoni *eggs and 189 were negative for both stool examinations.

Urine filtration detected only 13 children excreting eggs of *Schistosoma haematobium *(2.7%). Among them, 11 were also positive for *S. mansoni*. The stool examination showed the presence of 31 other intestinal parasitic infections (Ascaris and Trichuris). Patients negative for *S. mansoni *and carriers of another intestinal (12 subjects) or urinary parasite (2 subjects) were excluded of the analysis.

In total, 511 children were included in the analysis (336 infected by *S. mansoni *(65.7%) and 175 (34.3%) not infected). The prevalence increased as a function of age, reaching 80% in children older than 11 years with > 30% of children presenting with high schistosome loads, i.e. > 1,000 eggs/ 1 g of stools.

The malaria index was 11.2% in September, 8.1 % in October, 8.6 % in November and 5% in February. Malaria attacks were detected by passive case detection, ie as out-patients at the dispensary. Among 262 of the cohort patients consulting for fever, 107 cases (40.8 %) were attributed to a malaria attack, according to the criteria described. Only 10 children underwent two malaria attacks. In total, 18.9 % (97/511) of the children included in the analysis presented with a malaria attack at least once.

Chemoprophylaxis for children is not recommended by the National Control Programme, but 7.9 % of the parents reported that they were giving a chemoprophylactic treatment to their children. 58.9 % stated they used a mosquito bednet. These proportions were the same in the two groups of children, infected by *S. mansonii*or not. However, the malaria attack prevalence was also the same whether or not the children were sleeping under a mosquito bednet.

The incidence rate of malaria attacks was 20.2 % (68/336) in the group of children concomitantly infected by *S. mansoni *and 16.6% (29/175) in those non-infected (p = 0.40). The malarial incidence, however, varied depending on the load of eggs. It was high in all groups, but the highest (26.6 %) in those carrying the highest worm loads (> 1,000), except in subjects presenting medium loads (>100 and < 400 eggs/g of stools) where malarial incidence was lower than in *S. mansoni *negative individuals (9.4 %). Sex and age had no significant influence, which is not surprising since this is a mesoendemic area. Using a logistic regression model and taking the negative group as reference, the difference was significant with the group carrying a high load of eggs (RR = 2.24 (1.2 – 4.2) (Table I). Among all *S. mansoni *positive individuals, and using the group with medium egg load as reference (lower incidence of malaria attacks), the malaria incidence was significantly increased for low *S. mansoni *egg carriage (1–100) (RR = 2.51 (1.05–6) as well as high *S. mansoni *egg carriage (>1,000) RR = 3.12(1.33–7.29).

**Table 1:** Logistic regression model for malaria attack adjusting for sex, age and load in eggs of *S. mansoni*/g of stools, n = 511, Richard Toll, Senegal, 1999

	Odds Ratio	P>|z|	[95% Conf. Interval]
Sex*	.72	0.16	.46–1.13
Egg's load**			
1–100 (n = 99)	1.82	0.06	.97 – 3.42
101–400 (n = 73)	.72	0.46	.31 – 1.70
401–1000 (n = 55)	1.46	0.35	.66 – 3.25
> 1000 (n = 109)	2.24	0.01	1.20–4.20
Age	.99	0.79	.92–1.07

*Female, ** Non-infected children as reference, n = number of subjects in each group

Out of 10 children who presented two malaria attacks, nine were infected by S.m.; four presented a schistosome load higher than 1,000 eggs/g of stools, and three > 400 and <1,000.

**Figure 1:**
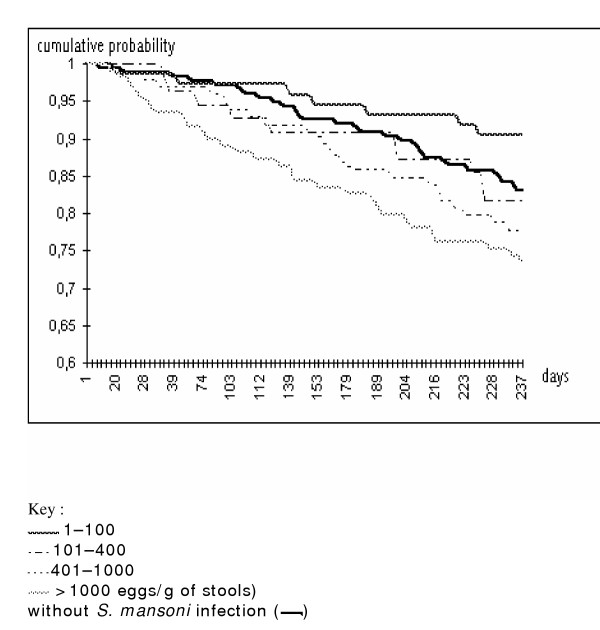
Probability of not having had a malaria attack in children presenting *S. mansoni *infection or without *S. mansoni *infection

The cumulative incidence of malaria attacks based on the Kaplan-Meier analysis according to the day of follow-up shows that the difference between subjects carrying high *S. mansoni *loads and other groups increased over time during the follow-up (Fig 1). This difference is particularly clear over the first ten weeks.

## Discussion

The high prevalence of *S. mansoni *and the low prevalence of urinary schistosomiasis in the area studied are in agreement with previous reports [4,6,7]. The prevalence of intestinal helminths, such as Ascaris and hookworms, was also low. The high prevalence of *S. mansoni*, together with a low prevalence of other helminthic infections, including *S. haematobium*, make this area suitable for studying the influence of *S. mansoni *upon *P. falciparum *infection. Malarial indices were consistently lower than 20 %, in agreement with previous studies which classified this northern Sahel area as meso-endemic [8,9]. Entomological studies suggest that this low endemicity could to be due to the dominance of *Anopheles pharoensis*, which is a poor vector in view of its short life-expectancy [10,11].

The analysis of the incidence of the malaria attacks by amount of parasite load in *S. mansoni *eggs suggests a more complex mechanism than a simple linear link between the frequency of malaria infection and the degree of infestation by *S. mansoni*. Indeed, the data show a greater rate of malaria attacks in children with either a high load (> 400 and > 1,000) or a low load of eggs (1–100), whereas a lower attack rate was observed in children presenting a medium egg load (>100 and <400 eggs/g), although this opposite trend did not reach significance.

Protection against falciparum malaria has been found to be associated with the preferential production of the cytophilic classes, IgG1 and IgG3, of antibodies [12], this being related to their ability to cooperate with blood monocytes in an ADCC-like (Antibody-Dependant Cellular cytotoxicity) mechanism [13]. Conversely, the very long delay needed to reach a state of protection was associated with the preferential production of non-cytophilic classes of antibodies, such as IgG2, IgG4 and IgM [13]. This also immediately raised the question of why the immune response to falciparum malaria is channeled to non-cytophilic classes in children and led to formulate the hypothesis that it could be related to helminthic co-infections, which are known to induce a Th2-like type of response. Indeed, children are prone to much higher helminthic loads than are adults. In Madagascar, a study conducted in children carrying intestinal helminths and treated with levamisole, suggested after a two-years follow-up, that a three-fold decrease in malaria attack rate was induced in helminth-treated subjects, as compared to non-treated paired controls [2]. These initial results were confirmed by more recent studies in which levamisole was not used [3,14].

In the present study, this observation has been confirmed with yet another worm infection, *S mansoni*, but only for those having the highest loads. The opposite effect was found in individuals carrying medium schistosome infections. As immunological studies could not be performed, one can only speculate about this biphasic effect. The production of cytophilic classes against malaria requires that the T-cells helper effect is provided by Th1 type of T-cells, however cytokines produced by T-cells specific for schistosome eggs or worms can influence responses to malaria. It has been reported that, during the first phase of *S. mansoni *infection, T-cells are stimulated which secrete cytokines belonging, in majority, to the Th1 type. The switch towards Th2 type cytokines occurs later on and is dependent on egg production [15]. In the case of this study, medium grade egg deposition may not be sufficient to modify the initial Th1 type of response, which would be dominant, contribute to accentuate the Th1 response to malaria and, therefore, to increase protection against malaria. Conversely, in subjects with high egg production, Th2 would become dominant and contribute to drive the antimalarial immune response towards non-cytophilic classes. An alternative hypothesis could be that lower egg-output actually reflects strong granuloma formation which, itself, is Th1 related, and conversely the high egg-output would indicate a Th2 type of response. Whereas this may account for the difference observed between medium and high worm load, it still does not provide an explanation for the increased susceptibility to malaria in the group excreting the lowest number of *S. mansoni *eggs.

Obviously, further studies, particularly of T-cell responses to both malarial and schistosome antigens are required to sort out this issue.

## Conclusions

Helminthic infections are a fact of life in malaria endemic areas and their influence on the course of infection and the epidemiology of malaria is a fascinating though neglected area of research. This study shows that *S. mansoni *infection can increase the susceptibility to malaria in subjects excreting high schistosome egg loads.

## Contribution of authors

CS, PM, JA, PC, MD were responsible for field and laboratory studies. JYL was responsible for the statistical analysis. PD inspired and designed the study
